# The genome sequence of a sawfly, *Abia candens *Konow, 1887

**DOI:** 10.12688/wellcomeopenres.23449.1

**Published:** 2025-01-10

**Authors:** Andrew Cunningham, Andrew Halstead, Natural History Museum Genome Acquisition Lab, Darwin Tree of Life Barcoding collective, Wellcome Sanger Institute Tree of Life Management, Samples and Laboratory team, Wellcome Sanger Institute Scientific Operations: Sequencing Operations, Wellcome Sanger Institute Tree of Life Core Informatics team, Tree of Life Core Informatics collective, Darwin Tree of Life Consortium

**Affiliations:** 1 Independent researcher, Tiverton, Devon, England, UK; 2 Independent researcher, Knaphill, Woking, Surrey, England, UK

**Keywords:** Abia candens, sawfly, genome sequence, chromosomal, Hymenoptera

## Abstract

We present a genome assembly from an individual male specimen of *Abia candens* (sawfly; Arthropoda; Insecta; Hymenoptera; Cimbicidae). The genome sequence has a total length of 261.00 megabases. Most of the assembly (82.7%) is scaffolded into 16 chromosomal pseudomolecules. The mitochondrial genome has also been assembled and is 19.72 kilobases in length.

## Species taxonomy

Eukaryota; Opisthokonta; Metazoa; Eumetazoa; Bilateria; Protostomia; Ecdysozoa; Panarthropoda; Arthropoda; Mandibulata; Pancrustacea; Hexapoda; Insecta; Dicondylia; Pterygota; Neoptera; Endopterygota; Hymenoptera; Tenthredinoidea; Cimbicidae; *Abia*; *Abia candens* Konow, 1887 (NCBI:txid362089)

## Background

*Abia candens* is a broad-bodied sawfly species of 9–12 mm in length. It is black with metallic purplish-green reflections and transparent wings with a dark mark under the stigma. As with other Cimbicidae sawflies, it has clubbed antennae. It is of local occurrence throughout Britain and has a scattered distribution elsewhere in Europe ( GBIF Secretariat, 2024). In Britain, the population is largely female with adults appearing in midsummer ( Quinlan & Gauld, 1981).

The larvae feed on the foliage of *Knautia arvensis* and *Succisa pratensis* ( Lacourt, 2020). *A. candens* has two generations a year.

The genome of the sawfly *Abia candens* was sequenced as part of the Darwin Tree of Life Project, a collaborative effort to sequence all named eukaryotic species in the Atlantic Archipelago of Britain and Ireland ( Blaxter *et al.*, 2022).

## Genome sequence report

**Figure 1.**
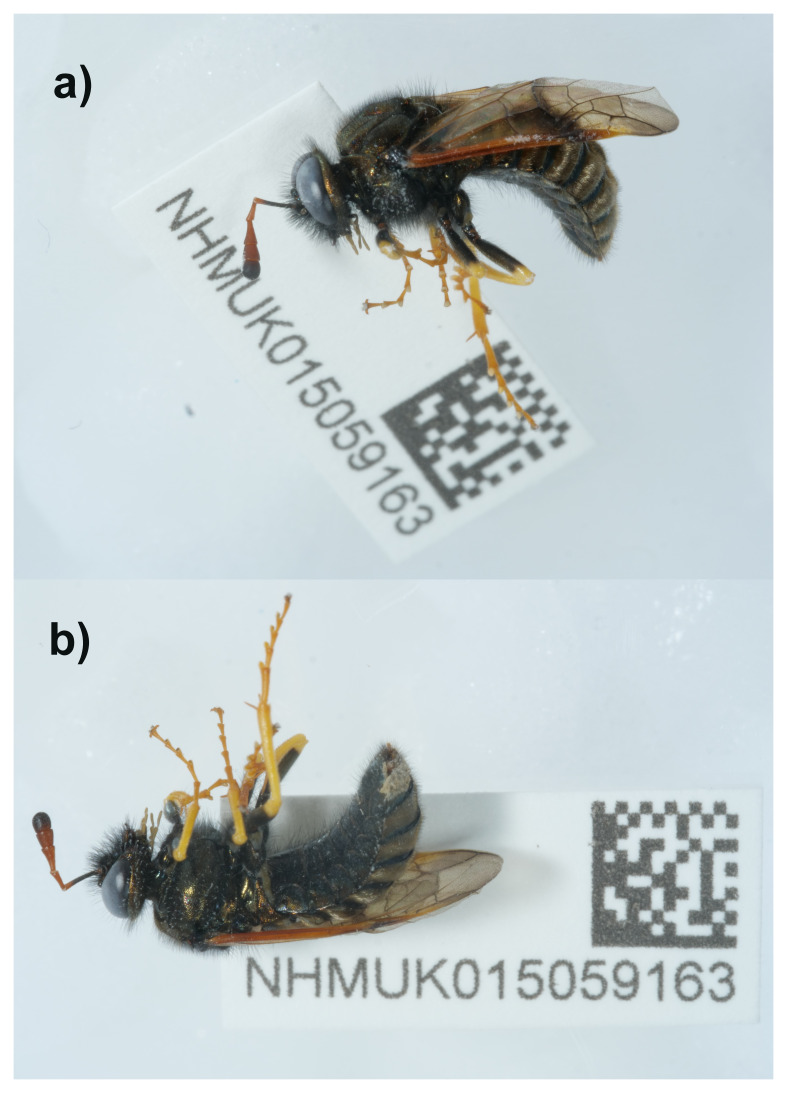
Photographs of the *Abia candens* (iyAbiCand1) specimen used for genome sequencing.

The genome of *Abia candens* ( Figure 1) was sequenced using Pacific Biosciences single-molecule HiFi long reads, generating a total of 8.39 Gb (gigabases) from 0.90 million reads, providing an estimated 38-fold coverage. Primary assembly contigs were scaffolded with chromosome conformation Hi-C data, which produced 147.06 Gb from 973.91 million reads. Specimen and sequencing details are summarised in Table 1.

**Table 1.** Specimen and sequencing data for *Abia candens*.

Project information			
**Study title**	Abia candens		
**Umbrella BioProject**	PRJEB66085		
**Species**	*Abia candens*		
**BioSample**	SAMEA112964485		
**NCBI taxonomy ID**	362089		
Specimen information			
**Technology**	**ToLID**	**BioSample accession**	**Organism part**
**PacBio long read sequencing**	iyAbiCand1	SAMEA112975685	Whole organism
**Hi-C sequencing**	iyAbiCand1	SAMEA112975685	Whole organism
Sequencing information			
**Platform**	**Run accession**	**Read count**	**Base count (Gb)**
**Hi-C Illumina NovaSeq 6000**	ERR12071284	9.74e+08	147.06
**PacBio Revio**	ERR12055606	9.01e+05	8.39

Assembly errors, including 43 missing joins or mis-joins, were corrected by manual curation. This reduced the scaffold number by 6.77% and increased the scaffold N50 by 3.91%. The final assembly has a total length of 261.00 Mb in 371 sequence scaffolds, with 262 gaps, and a scaffold N50 of 14.1 Mb ( Table 2).

**Table 2.** Genome assembly data for *Abia candens*, iyAbiCand1.1.

Genome assembly		
Assembly name	iyAbiCand1.1	
Assembly accession	GCA_964212075.1	
Span (Mb)	261.00	
Number of contigs	634	
Number of scaffolds	371	
Longest scaffold (Mb)	20.45	
Assembly metrics *		*Benchmark*
Contig N50 length (Mb)	1.2	*≥ 1 Mb*
Scaffold N50 length (Mb)	14.1	*= chromosome N50*
Consensus quality (QV)	56.6	*≥ 40*
*k*-mer completeness	99.99%	*≥ 95%*
lineage: hymenoptera_odb10	C:95.5%[S:95.2%,D:0.3%], F:1.4%,M:3.1%,n:5,991	*S > 90%, D < 5%*
Percentage of assembly mapped to chromosomes	82.7%	*≥ 90%*
Sex chromosomes	none	*localised homologous pairs*
Organelles	Mitochondrial genome: 19.72 kb	*complete single alleles*

* Assembly metric benchmarks are adapted from Rhie *et al.* (2021) and the Earth BioGenome Project Report on Assembly Standards September 2024.

**Figure 2.**
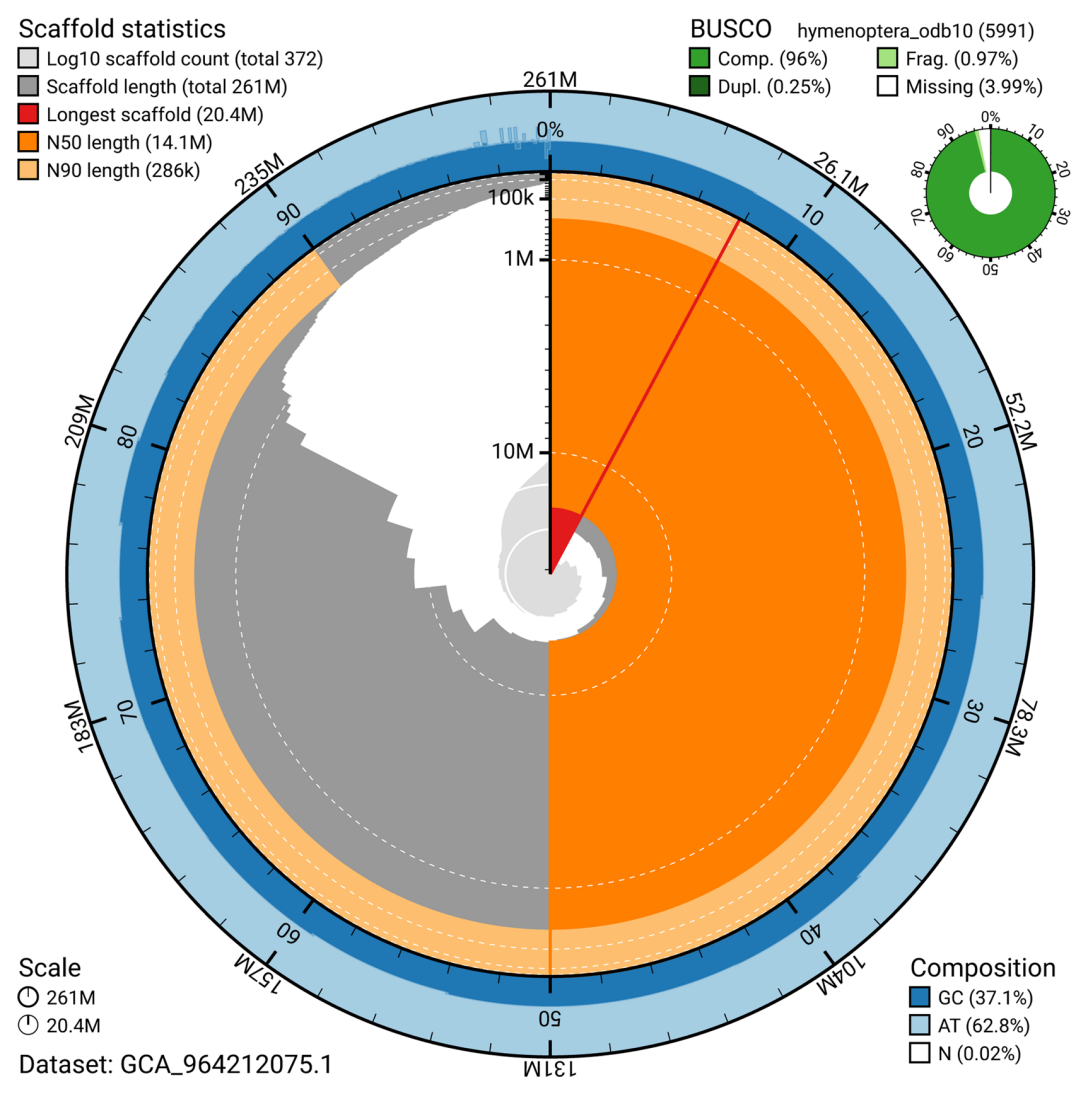
Genome assembly of *Abia candens*, iyAbiCand1.1: metrics. The BlobToolKit snail plot provides an overview of assembly metrics and BUSCO gene completeness. The circumference represents the length of the whole genome sequence, and the main plot is divided into 1,000 bins around the circumference. The outermost blue tracks display the distribution of GC, AT, and N percentages across the bins. Scaffolds are arranged clockwise from longest to shortest and are depicted in dark grey. The longest scaffold is indicated by the red arc, and the deeper orange and pale orange arcs represent the N50 and N90 lengths. A light grey spiral at the centre shows the cumulative scaffold count on a logarithmic scale. A summary of complete, fragmented, duplicated, and missing BUSCO genes in the hymenoptera_odb10 set is presented at the top right. An interactive version of this figure is available at https://blobtoolkit.genomehubs.org/view/GCA_964212075.1/dataset/GCA_964212075.1/snail.

**Figure 3.**
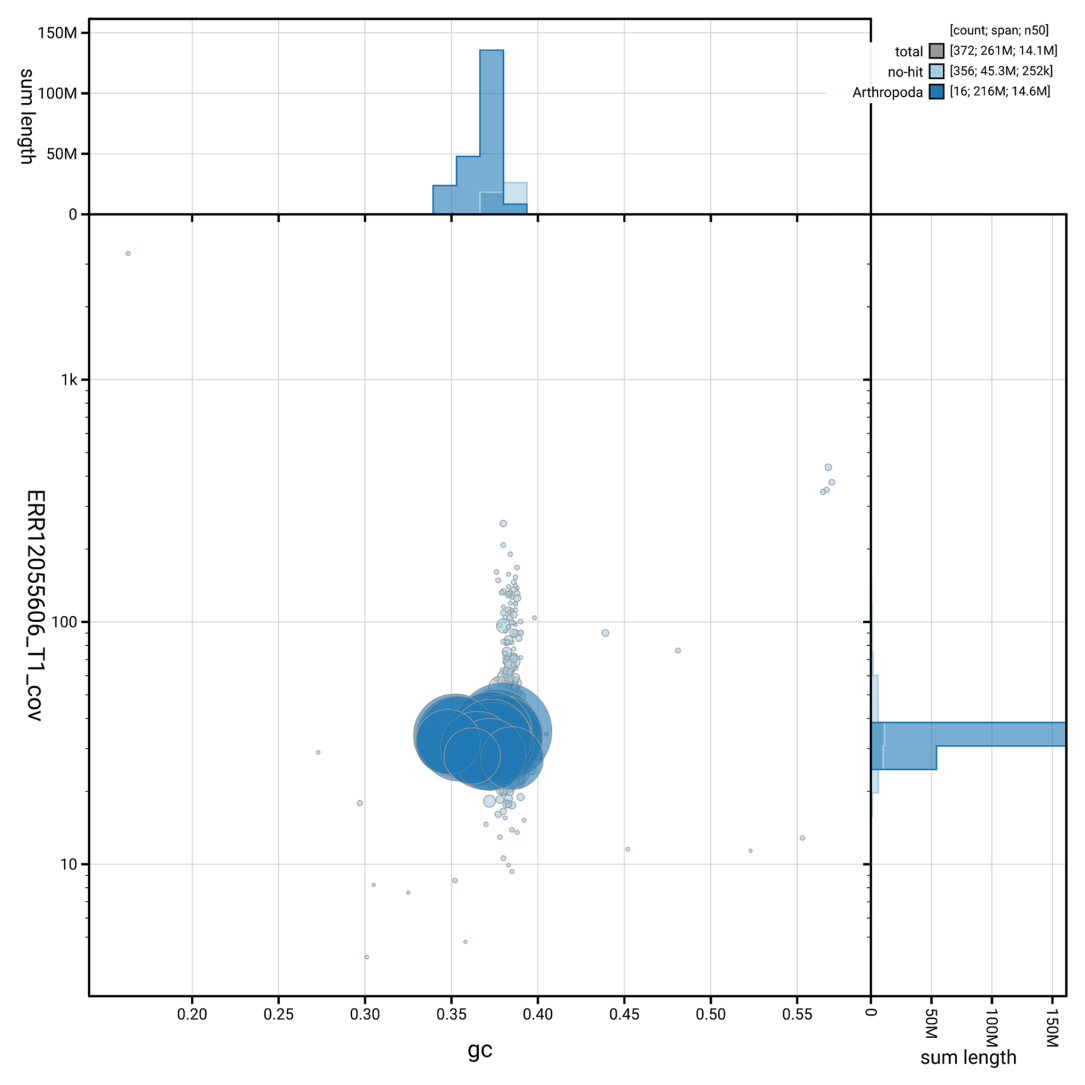
Genome assembly of *Abia candens*, iyAbiCand1.1: BlobToolKit GC-coverage plot showing sequence coverage (vertical axis) and GC content (horizontal axis). The circles represent scaffolds, with the size proportional to scaffold length and the colour representing phylum membership. The histograms along the axes display the total length of sequences distributed across different levels of coverage and GC content. An interactive version of this figure is available at https://blobtoolkit.genomehubs.org/view/GCA_964212075.1/dataset/GCA_964212075.1/blob.

**Figure 4.**
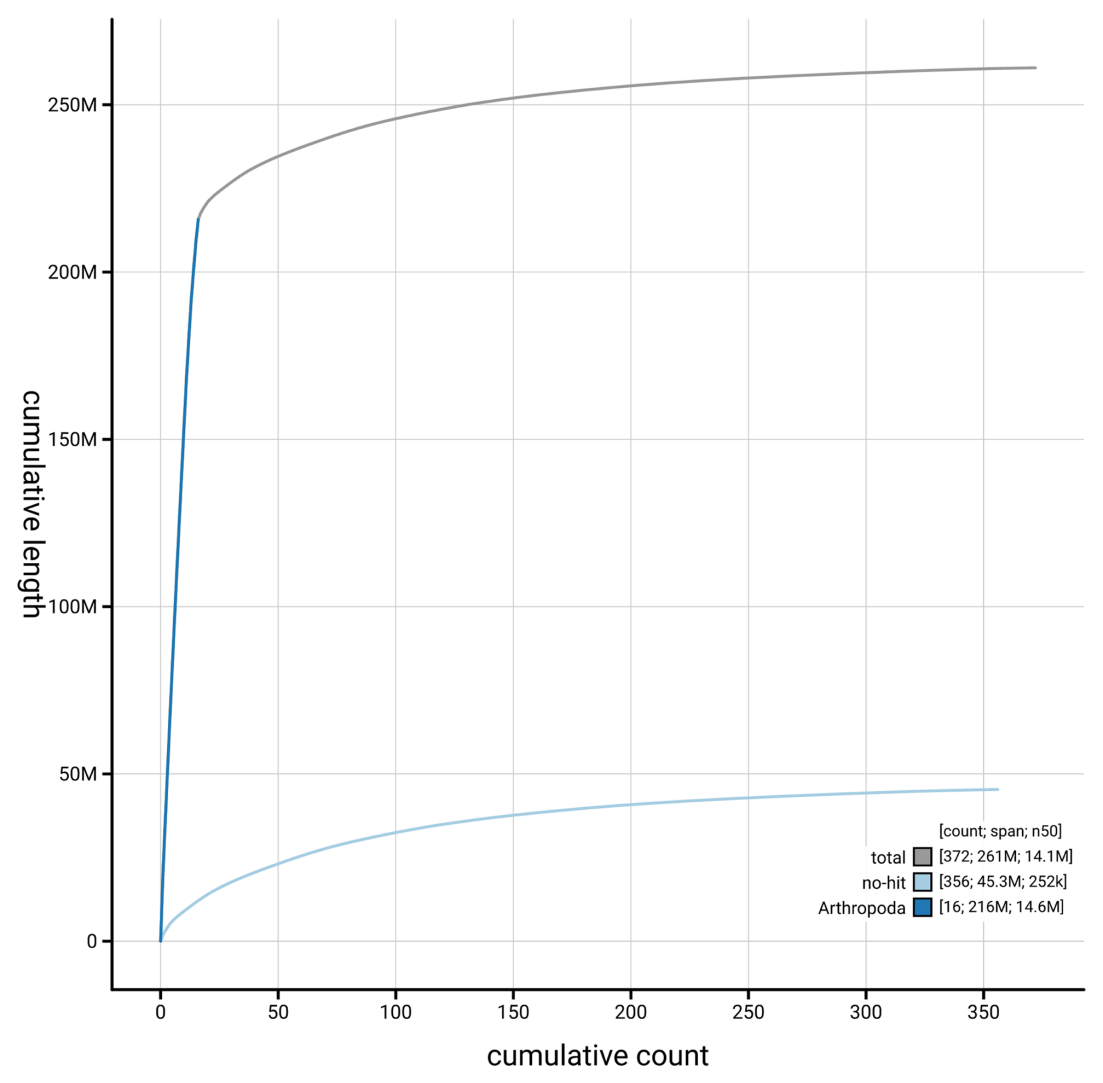
Genome assembly of *Abia candens* iyAbiCand1.1: BlobToolKit cumulative sequence plot. The grey line shows cumulative length for all scaffolds. Coloured lines show cumulative lengths of scaffolds assigned to each phylum using the buscogenes taxrule. An interactive version of this figure is available at https://blobtoolkit.genomehubs.org/view/GCA_964212075.1/dataset/GCA_964212075.1/cumulative.

The snail plot in Figure 2 provides a summary of the assembly statistics, indicating the distribution of scaffold lengths and other assembly metrics. Figure 3 shows the distribution of scaffolds by GC proportion and coverage. Figure 4 presents a cumulative assembly plot, with separate curves representing different scaffold subsets assigned to various phyla, illustrating the completeness of the assembly.

**Figure 5.**
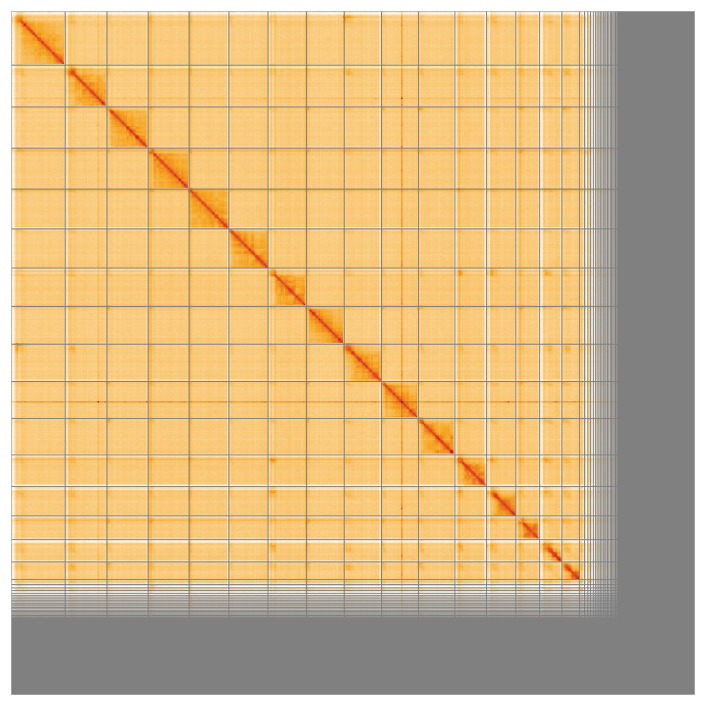
Genome assembly of *Abia candens* iyAbiCand1.1: Hi-C contact map of the iyAbiCand1.1 assembly, visualised using HiGlass. Chromosomes are shown in order of size from left to right and top to bottom. An interactive version of this figure may be viewed at https://genome-note-higlass.tol.sanger.ac.uk/l/?d=PbLB0FINQYenABwwbHohcw.

Most of the assembly sequence (82.7%) was assigned to 16 chromosomal-level scaffolds. These chromosome-level scaffolds, confirmed by the Hi-C data, are named in order of size ( Figure 5; Table 3). The assembly is a of a haploid male specimen.

**Table 3.** Chromosomal pseudomolecules in the genome assembly of *Abia candens*, iyAbiCand1.

INSDC accession	Name	Length (Mb)	GC%
OZ125706.1	1	20.45	38.0
OZ125707.1	2	15.84	37.0
OZ125708.1	3	15.7	37.5
OZ125709.1	4	15.54	38.0
OZ125710.1	5	15.1	35.5
OZ125711.1	6	14.65	38.0
OZ125712.1	7	14.82	35.0
OZ125713.1	8	14.33	37.5
OZ125714.1	9	14.12	37.5
OZ125715.1	10	14.03	35.5
OZ125716.1	11	13.82	37.5
OZ125717.1	12	11.92	36.5
OZ125718.1	13	11.22	37.0
OZ125719.1	14	8.99	35.0
OZ125720.1	15	8.43	38.5
OZ125721.1	16	6.76	36.0
OZ125722.1	MT	0.02	16.5

The mitochondrial genome was also assembled and can be found as a contig within the multifasta file of the genome submission, and as a separate fasta file with accession OZ125722.1.

The final assembly has a Quality Value (QV) of 56.6 and *k*-mer completeness of 99.99%. BUSCO (v5.4.3) analysis using the hymenoptera_odb10 reference set ( *n* = 5,991) indicated a completeness score of 95.5% (single = 95.2%, duplicated = 0.3%). The assembly achieves the EBP reference standard of 6.7.56.6. Other quality metrics are given in Table 2. 

## Methods

### Sample acquisition and DNA barcoding

An adult male specimen of *Abia candens* (specimen ID NHMUK015059163, ToLID iyAbiCand1) was collected from Foulden, England, United Kingdom (latitude 52.56, longitude 0.6) on 2022-07-04. The specimen was collected by Andrew Cunningham and identified by Andrew Halstead (both Dipterists Forum), and then preserved by dry freezing at – 80 °C.

The initial identification was verified by an additional DNA barcoding process according to the framework developed by Twyford *et al*. (2024). A small sample was dissected from the specimens and stored in ethanol, while the remaining parts were shipped on dry ice to the Wellcome Sanger Institute (WSI). The tissue was lysed, the COI marker region was amplified by PCR, and amplicons were sequenced and compared to the BOLD database, confirming the species identification ( Crowley *et al.*, 2023). Following whole genome sequence generation, the relevant DNA barcode region was also used alongside the initial barcoding data for sample tracking at the WSI ( Twyford *et al.*, 2024). The standard operating procedures for Darwin Tree of Life barcoding have been deposited on protocols.io ( Beasley *et al.*, 2023).

### Nucleic acid extraction

The workflow for high molecular weight (HMW) DNA extraction at the WSI Tree of Life Core Laboratory includes a sequence of procedures: sample preparation and homogenisation, DNA extraction, fragmentation and purification. Detailed protocols are available on protocols.io ( Denton *et al.*, 2023b). The iyAbiCand1 sample was prepared for DNA extraction by weighing and dissecting it on dry ice ( Jay *et al.*, 2023) and was homogenised using a PowerMasher II tissue disruptor ( Denton *et al.*, 2023a).

HMW DNA was extracted in the WSI Scientific Operations core using the Automated MagAttract v2 protocol ( Oatley *et al.*, 2023). The DNA was sheared into an average fragment size of 12–20 kb in a Megaruptor 3 system ( Bates *et al.*, 2023). Sheared DNA was purified by solid-phase reversible immobilisation, using AMPure PB beads to eliminate shorter fragments and concentrate the DNA ( Strickland *et al.*, 2023). The concentration of the sheared and purified DNA was assessed using a Nanodrop spectrophotometer and Qubit Fluorometer using the Qubit dsDNA High Sensitivity Assay kit. Fragment size distribution was evaluated by running the sample on the FemtoPulse system.

### Hi-C preparation

Tissue from the iyAbiCand1 sample was processed at the WSI Scientific Operations core, using the Arima-HiC v2 kit. Tissue (stored at –80 °C) was fixed, and the DNA crosslinked using a TC buffer with 22% formaldehyde. After crosslinking, the tissue was homogenised using the Diagnocine Power Masher-II and BioMasher-II tubes and pestles. Following the kit manufacturer's instructions, crosslinked DNA was digested using a restriction enzyme master mix. The 5’-overhangs were then filled in and labelled with biotinylated nucleotides and proximally ligated. An overnight incubation was carried out for enzymes to digest remaining proteins and for crosslinks to reverse. A clean up was performed with SPRIselect beads prior to library preparation.

### Library preparation and sequencing

Library preparation and sequencing were performed at the WSI Scientific Operations core. Pacific Biosciences HiFi circular consensus DNA sequencing libraries were prepared using the PacBio Express Template Preparation Kit v2.0 (Pacific Biosciences, California, USA) as per the manufacturer's instructions. The kit includes the reagents required for removal of single-strand overhangs, DNA damage repair, end repair/A-tailing, adapter ligation, and nuclease treatment. Library preparation also included a library purification step using AMPure PB beads (Pacific Biosciences, California, USA) and size selection step to remove templates shorter than 3 kb using AMPure PB modified SPRI. DNA concentration was quantified using the Qubit Fluorometer v2.0 and Qubit HS Assay Kit and the final library fragment size analysis was carried out using the Agilent Femto Pulse Automated Pulsed Field CE Instrument and gDNA 165kb gDNA and 55kb BAC analysis kit. Samples were sequenced using the Sequel IIe system (Pacific Biosciences, California, USA). The concentration of the library loaded onto the Sequel IIe was in the range 40–135 pM. The SMRT link software, a PacBio web-based end-to-end workflow manager, was used to set-up and monitor the run, as well as perform primary and secondary analysis of the data upon completion.

For Hi-C library preparation, DNA was fragmented to a size of 400 to 600 bp using a Covaris E220 sonicator. The DNA was then enriched, barcoded, and amplified using the NEBNext Ultra II DNA Library Prep Kit following manufacturers’ instructions. The Hi-C sequencing was performed using paired-end sequencing with a read length of 150 bp on an Illumina NovaSeq 6000 instrument.

### Genome assembly, curation and evaluation

***Assembly***

The HiFi reads were first assembled using Hifiasm ( Cheng *et al.*, 2021). The Hi-C reads were mapped to the primary contigs using bwa-mem2 ( Vasimuddin *et al.*, 2019). The contigs were further scaffolded using the provided Hi-C data ( Rao *et al.*, 2014) in YaHS ( Zhou *et al.*, 2023) using the --break option for handling potential misassemblies. The scaffolded assemblies were evaluated using Gfastats ( Formenti *et al.*, 2022), BUSCO ( Manni *et al.*, 2021) and MERQURY.FK ( Rhie *et al.*, 2020).

The mitochondrial genome was assembled using OatK ( Zhou *et al.*, 2024) and MitoHiFi ( Uliano-Silva *et al.*, 2023), which runs MitoFinder ( Allio *et al.*, 2020) and uses these annotations to select the final mitochondrial contig and to ensure the general quality of the sequence.

***Assembly curation***

The assembly was decontaminated using the Assembly Screen for Cobionts and Contaminants (ASCC) pipeline (article in preparation). Flat files and maps used in curation were generated in TreeVal ( Pointon *et al.*, 2023). Manual curation was primarily conducted using PretextView ( Harry, 2022), with additional insights provided by JBrowse2 ( Diesh *et al.*, 2023) and HiGlass ( Kerpedjiev *et al.*, 2018). Scaffolds were visually inspected and corrected as described by Howe *et al*. (2021). Any identified contamination, missed joins, and mis-joins were corrected, and duplicate sequences were tagged and removed. The curation process is documented at https://gitlab.com/wtsi-grit/rapid-curation (article in preparation).

***Evaluation of the final assembly***

The final assembly was post-processed and evaluated using the three Nextflow ( Di Tommaso *et al.*, 2017) DSL2 pipelines: sanger-tol/readmapping ( Surana *et al.*, 2023a), sanger-tol/genomenote ( Surana *et al.*, 2023b), and sanger-tol/blobtoolkit ( Muffato *et al.*, 2024). The readmapping pipeline aligns the Hi-C reads using bwa-mem2 ( Vasimuddin *et al.*, 2019) and combines the alignment files with SAMtools ( Danecek *et al.*, 2021). The genomenote pipeline converts the Hi-C alignments into a contact map using BEDTools ( Quinlan & Hall, 2010) and the Cooler tool suite ( Abdennur & Mirny, 2020). The contact map is visualised in HiGlass ( Kerpedjiev *et al.*, 2018). This pipeline also computes *k*-mer completeness and QV consensus quality values with FastK and MERQURY.FK, and runs BUSCO ( Manni *et al.*, 2021) to assess completeness.

The blobtoolkit pipeline is a Nextflow port of the previous Snakemake Blobtoolkit pipeline ( Challis *et al.*, 2020). It aligns the PacBio reads in SAMtools and minimap2 ( Li, 2018) and generates coverage tracks for regions of fixed size. In parallel, it queries the GoaT database ( Challis *et al.*, 2023) to identify all matching BUSCO lineages to run BUSCO ( Manni *et al.*, 2021). For the three domain-level BUSCO lineages, the pipeline aligns the BUSCO genes to the UniProt Reference Proteomes database ( Bateman *et al.*, 2023) with DIAMOND blastp ( Buchfink *et al.*, 2021). The genome is also divided into chunks according to the density of the BUSCO genes from the closest taxonomic lineage, and each chunk is aligned to the UniProt Reference Proteomes database using DIAMOND blastx. Genome sequences without a hit are chunked using seqtk and aligned to the NT database with blastn ( Altschul *et al.*, 1990). The blobtools suite combines all these outputs into a blobdir for visualisation.

The genome evaluation pipelines were developed using nf-core tooling ( Ewels *et al.*, 2020) and MultiQC ( Ewels *et al.*, 2016), relying on the Conda package manager, the Bioconda initiative ( Grüning *et al.*, 2018), the Biocontainers infrastructure ( da Veiga Leprevost *et al.*, 2017), as well as the Docker ( Merkel, 2014) and Singularity ( Kurtzer *et al.*, 2017) containerisation solutions.

Table 4 contains a list of relevant software tool versions and sources.

**Table 4.** Software tools: versions and sources.

Software tool	Version	Source
BEDTools	2.30.0	https://github.com/arq5x/bedtools2
BLAST	2.14.0	ftp://ftp.ncbi.nlm.nih.gov/blast/executables/blast+/
BlobToolKit	4.3.7	https://github.com/blobtoolkit/blobtoolkit
BUSCO	5.4.3 and 5.5.0	https://gitlab.com/ezlab/busco
bwa-mem2	2.2.1	https://github.com/bwa-mem2/bwa-mem2
Cooler	0.8.11	https://github.com/open2c/cooler
DIAMOND	2.1.8	https://github.com/bbuchfink/diamond
fasta_windows	0.2.4	https://github.com/tolkit/fasta_windows
FastK	427104ea91c78c3b8b8b49f1a7d6bbeaa869ba1c	https://github.com/thegenemyers/FASTK
Gfastats	1.3.6	https://github.com/vgl-hub/gfastats
GoaT CLI	0.2.5	https://github.com/genomehubs/goat-cli
Hifiasm	0.16.1	https://github.com/chhylp123/hifiasm
HiGlass	44086069ee7d4d3f6f3f0012569789ec138f42b84 aa44357826c0b6753eb28de	https://github.com/higlass/higlass
Merqury.FK	d00d98157618f4e8d1a9190026b19b471055b2 2e	https://github.com/thegenemyers/MERQURY.FK
MitoHiFi	2	https://github.com/marcelauliano/MitoHiFi
MultiQC	1.14, 1.17, and 1.18	https://github.com/MultiQC/MultiQC
NCBI Datasets	15.12.0	https://github.com/ncbi/datasets
Nextflow	23.04.0-5857	https://github.com/nextflow-io/nextflow
OATK	1	https://github.com/c-zhou
PretextView	0.2.5	https://github.com/sanger-tol/PretextView
samtools	1.16.1, 1.17, and 1.18	https://github.com/samtools/samtools
sanger-tol/ascc	-	https://github.com/sanger-tol/ascc
sanger-tol/genomenote	1.1.1	https://github.com/sanger-tol/genomenote
sanger-tol/readmapping	1.2.1	https://github.com/sanger-tol/readmapping
Seqtk	1.3	https://github.com/lh3/seqtk
Singularity	3.9.0	https://github.com/sylabs/singularity
TreeVal	1.0.0	https://github.com/sanger-tol/treeval
YaHS	1.1a.2	https://github.com/c-zhou/yahs

### Wellcome Sanger Institute – Legal and Governance

The materials that have contributed to this genome note have been supplied by a Darwin Tree of Life Partner. The submission of materials by a Darwin Tree of Life Partner is subject to the **‘Darwin Tree of Life Project Sampling Code of Practice’**, which can be found in full on the Darwin Tree of Life website here. By agreeing with and signing up to the Sampling Code of Practice, the Darwin Tree of Life Partner agrees they will meet the legal and ethical requirements and standards set out within this document in respect of all samples acquired for, and supplied to, the Darwin Tree of Life Project.

Further, the Wellcome Sanger Institute employs a process whereby due diligence is carried out proportionate to the nature of the materials themselves, and the circumstances under which they have been/are to be collected and provided for use. The purpose of this is to address and mitigate any potential legal and/or ethical implications of receipt and use of the materials as part of the research project, and to ensure that in doing so we align with best practice wherever possible. The overarching areas of consideration are:

ߦ Ethical review of provenance and sourcing of the material

ߦ Legality of collection, transfer and use (national and international)

Each transfer of samples is further undertaken according to a Research Collaboration Agreement or Material Transfer Agreement entered into by the Darwin Tree of Life Partner, Genome Research Limited (operating as the Wellcome Sanger Institute), and in some circumstances other Darwin Tree of Life collaborators.

## Data Availability

European Nucleotide Archive: Abia candens. Accession number PRJEB66085; https://identifiers.org/ena.embl/PRJEB66085. The genome sequence is released openly for reuse. The *Abia candens* genome sequencing initiative is part of the Darwin Tree of Life (DToL) project. All raw sequence data and the assembly have been deposited in INSDC databases. The genome will be annotated using available RNA-Seq data and presented through the Ensembl pipeline at the European Bioinformatics Institute. Raw data and assembly accession identifiers are reported in Table 1 and Table 2. Metadata for specimens, BOLD barcode results, spectra estimates, sequencing runs, contaminants and pre-curation assembly statistics are given at https://links.tol.sanger.ac.uk/species/362089.

## Author information

Members of the Natural History Museum Genome Acquisition Lab are listed here: https://doi.org/10.5281/zenodo.12159242.

Members of the Darwin Tree of Life Barcoding collective are listed here: https://doi.org/10.5281/zenodo.12158331.

Members of the Wellcome Sanger Institute Tree of Life Management, Samples and Laboratory team are listed here: https://doi.org/10.5281/zenodo.12162482.

Members of Wellcome Sanger Institute Scientific Operations: Sequencing Operations are listed here: https://doi.org/10.5281/zenodo.12165051.

Members of the Wellcome Sanger Institute Tree of Life Core Informatics team are listed here: https://doi.org/10.5281/zenodo.12160324.

Members of the Tree of Life Core Informatics collective are listed here: https://doi.org/10.5281/zenodo.12205391.

Members of the Darwin Tree of Life Consortium are listed here: https://doi.org/10.5281/zenodo.4783558.
