# Supplementary material for: The genome sequence of a sawfly, Abia candens Konow, 1887
Source: Wellcome Open Res. 2025 Jan 10;10:2. [Version 1] doi: 10.12688/wellcomeopenres.23449.1 (PMC11795027; doi:10.12688/wellcomeopenres.23449.1)
Supplement: Peer review history

# Reviewer report: Reviewer response for version 1

**DOI:** 10.21956/wellcomeopenres.25859.r117948

**Published:** 2025-02-04

**Authors:** Danilo Trabudo do Amaral

**Affiliations:** 1 Universidade Federal do ABC (UFABC), São Paulo, Brazil

It was a pleasure to review this work. The data is well explained, and the methodology is very detailed, making the study easy to follow and reproducible. I just suggest adding a quick description of the mitochondrial genome characteristics to indicate whether it follows the standard mtDNA structure for insects or not. This small addition could be useful for future comparative studies.

 Overall, this is a very well-conducted study, and I appreciate the opportunity to review it.

Are sufficient details of methods and materials provided to allow replication by others?

Yes

Is the rationale for creating the dataset(s) clearly described?

Yes

Are the datasets clearly presented in a useable and accessible format?

Yes

Are the protocols appropriate and is the work technically sound?

Yes

Reviewer Expertise:

Bioinformatic

I confirm that I have read this submission and believe that I have an appropriate level of expertise to confirm that it is of an acceptable scientific standard.

# Reviewer report: Reviewer response for version 1

**DOI:** 10.21956/wellcomeopenres.25859.r117696

**Published:** 2025-01-24

**Authors:** Nicolas Pollet

**Affiliations:** 1 Université Paris-Saclay, Orsay, France

Andrew Cunningham and colleagues present a chromosome-level genome sequence assembly for the the sawfly *Abia candens*. The genome is of high-quality and will provide great genetic resources for the global scientific community. I noticed that the mitochondrial genome sequence OZ125722.1 does not start at the standard position used for downstream annotations.

Are sufficient details of methods and materials provided to allow replication by others?

Yes

Is the rationale for creating the dataset(s) clearly described?

Yes

Are the datasets clearly presented in a useable and accessible format?

Yes

Are the protocols appropriate and is the work technically sound?

Yes

Reviewer Expertise:

Genomics Transcriptomics Evolution Biodiversity Metagenomics

I confirm that I have read this submission and believe that I have an appropriate level of expertise to confirm that it is of an acceptable scientific standard.
